# Evidence for inflammation in normal-appearing brain regions in patients with growing sporadic vestibular schwannoma: A PET study

**DOI:** 10.1093/noajnl/vdae094

**Published:** 2024-06-08

**Authors:** Bandar Alfaifi, Rainer Hinz, Alan Jackson, Andrea Wadeson, Omar N Pathmanaban, Charlotte Hammerbeck-Ward, Scott A Rutherford, Andrew T King, Daniel Lewis, David J Coope

**Affiliations:** Division of Informatics, Imaging and Data Sciences, School of Health Sciences, University of Manchester, Manchester, UK; Division of Informatics, Imaging and Data Sciences, School of Health Sciences, University of Manchester, Manchester, UK; Division of Informatics, Imaging and Data Sciences, School of Health Sciences, University of Manchester, Manchester, UK; Department of Neurosurgery, Manchester Centre for Clinical Neurosciences, Salford Royal NHS Foundation Trust, Manchester Academic Health Science Centre, Manchester, UK; Geoffrey Jefferson Brain Research Centre, University of Manchester, Manchester, UK; Department of Neurosurgery, Manchester Centre for Clinical Neurosciences, Salford Royal NHS Foundation Trust, Manchester Academic Health Science Centre, Manchester, UK; Geoffrey Jefferson Brain Research Centre, University of Manchester, Manchester, UK; Division of Cell Matrix Biology & Regenerative Medicine, School of Biological Sciences, University of Manchester, Manchester, UK; Department of Neurosurgery, Manchester Centre for Clinical Neurosciences, Salford Royal NHS Foundation Trust, Manchester Academic Health Science Centre, Manchester, UK; Department of Neurosurgery, Manchester Centre for Clinical Neurosciences, Salford Royal NHS Foundation Trust, Manchester Academic Health Science Centre, Manchester, UK; Geoffrey Jefferson Brain Research Centre, University of Manchester, Manchester, UK; Department of Neurosurgery, Manchester Centre for Clinical Neurosciences, Salford Royal NHS Foundation Trust, Manchester Academic Health Science Centre, Manchester, UK; Geoffrey Jefferson Brain Research Centre, University of Manchester, Manchester, UK; Division of Cardiovascular Sciences, School of Medical Sciences, University of Manchester, Manchester, UK; Department of Neurosurgery, Manchester Centre for Clinical Neurosciences, Salford Royal NHS Foundation Trust, Manchester Academic Health Science Centre, Manchester, UK; Geoffrey Jefferson Brain Research Centre, University of Manchester, Manchester, UK; Division of Neuroscience and Experimental Psychology, School of Biological Sciences, University of Manchester, Manchester, UK; Department of Neurosurgery, Manchester Centre for Clinical Neurosciences, Salford Royal NHS Foundation Trust, Manchester Academic Health Science Centre, Manchester, UK; Geoffrey Jefferson Brain Research Centre, University of Manchester, Manchester, UK; Division of Neuroscience and Experimental Psychology, School of Biological Sciences, University of Manchester, Manchester, UK

**Keywords:** inflammation, microglia, PET, TSPO, vestibular schwannoma

## Abstract

**Background:**

Nonauditory symptoms can be a prominent feature in patients with sporadic vestibular schwannoma (VS), but the cause of these symptoms is unknown. Inflammation is hypothesized to play a key role in the growth and symptomatic presentation of sporadic VS, and in this study, we investigated through translocator protein (TSPO) positron emission tomography (PET) whether inflammation occurred within the “normal appearing” brain of such patients and its association with tumor growth.

**Methods:**

Dynamic PET datasets from 15 patients with sporadic VS (8 static and 7 growing) who had been previously imaged using the TSPO tracer [^11^C](*R*)-PK11195 were included. Parametric images of [^11^C](*R*)-PK11195 binding potential (BP_ND_) and the distribution volume ratio (DVR) were derived and compared across VS growth groups within both contralateral and ipsilateral gray (GM) and white matter (WM) regions. Voxel-wise cluster analysis was additionally performed to identify anatomical regions of increased [^11^C](*R*)-PK11195 binding.

**Results:**

Compared with static tumors, growing VS demonstrated significantly higher cortical (GM, 1.070 vs. 1.031, *P* = .03) and whole brain (GM & WM, 1.045 vs. 1.006, *P* = .03) [^11^C](*R*)-PK11195 DVR values. The voxel-wise analysis supported the region-based analysis and revealed clusters of high TSPO binding within the precentral, postcentral, and prefrontal cortex in patients with growing VS.

**Conclusions:**

We present the first in vivo evidence of increased TSPO expression and inflammation within the brains of patients with growing sporadic VS. These results provide a potential mechanistic insight into the development of nonauditory symptoms in these patients and highlight the need for further studies interrogating the role of neuroinflammation in driving VS symptomatology.

**Key Points**

- Nonauditory symptoms can feature in patients with sporadic vestibular schwannoma (VS).
- Growing VS is associated with widespread TSPO upregulation within the normal brain.
- TSPO upregulation occurs within nonauditory and premotor frontal lobe regions.

**Importance of the Study**

Nonauditory symptoms can be a prominent feature in patients with sporadic vestibular schwannoma (VS), but the cause of these symptoms is unknown. Within this study we demonstrate for the first time in vivo using a dedicated PET tracer, that in patients with growing sporadic VS there is widespread translocator protein (TSPO) upregulation within normal-appearing brain regions, indicative of microglial activation. We furthermore demonstrate through a voxel-wise cluster analysis that this TSPO upregulation occurs within the nonauditory motor and premotor frontal lobe regions. This work provides an important, possible pathophysiological explanation for the nonaudiovestibular and neuropsychological symptoms experienced by patients with sporadic VS and a potential future therapeutic target (ie reversible inflammation) for these symptoms. These results furthermore highlight the need for further studies interrogating the role of neuroinflammation in patients with VS and other extra-axial CNS tumors.

Vestibular schwannoma (VS) are tumors that arise from Schwann cells surrounding the vestibulocochlear nerve.^1,2^ Although histologically benign, VS can cause considerable morbidity and impact on health-related quality of life. These tumors show a variable, sometimes unpredictable natural history with around 70% of untreated tumors showing volumetric growth at 3 years following diagnosis, with the remainder remaining stable or less commonly shrinking.^3^ The most common presenting symptom in VS is sensorineural hearing loss (SNHL), occurring in up to 95% of affected patients.^4,5^ In large tumors adjacent cranial nerves and brainstem can also become compressed, producing cranial nerve dysfunction, cerebellar symptoms, and hydrocephalus.^6^ Affected patients commonly report other distressing symptoms not directly attributable to the tumor’s location or size, such as reduced attention, decreases in executive function and psychomotor speed, worsening memory, fatigue, and depression.^7–11^

At present the cause of these pervasive nonauditory symptoms is not known but previous studies have shown widespread changes in WM fiber integrity, gray matter (GM) volume, and functional activity networks in auditory and nonauditory pathways in patients with VS.^7–9,12,13^ An emerging paradigm in VS pathogenesis is the role that inflammation may play in driving VS progression and growth.^1,2,14,15^ Growing VS are characterized by a dense infiltration of tumor-associated macrophages and VS growth is associated with increases in circulating pro-inflammatory mediators.^14,16^ An association between secreted candidate pro-inflammatory mediators, cochlear hair cell damage and SNHL in VS has also been described,^4,5^ but the extent to which inflammation also occurs within the brain of these patients and drives symptomatology is currently unknown.

In vivo, inflammation within the central nervous system (CNS) can be interrogated using dedicated positron emission tomography (PET) radiotracers that bind to the 18 kDa translocator protein (TSPO). TSPO is a mitochondrial protein and an inflammation biomarker that is significantly upregulated following microglia or macrophage activation.^17,18^ A candidate TSPO PET radiotracer is [^11^C](*R*)PK11195, and this tracer shows increased uptake in a wide range of acute and chronic neuroinflammatory and neurodegenerative conditions.^18–24^ In the only TSPO PET study in sporadic VS to date, our group previously demonstrated high [^11^C](*R*)PK11195 binding within growing VS and an association between tumor growth and the presence of TSPO-expressing macrophages within the tumor microenvironment.^2^ To date, however, there has been no study of TSPO upregulation or inflammation within the brains of patients with sporadic VS. The purpose of this new analysis, therefore, was to study changes in the normal-appearing brains for this cohort of patients imaged using dynamic [^11^C](*R*)PK11195 PET and interrogate the association between tumor growth and expression of TSPO within normal-appearing brain regions.

## Materials and Methods

### Study Participants

Datasets from 15 patients with either static (nongrowing) or growing unilateral sporadic VS were included in this study. All patients had been recruited prospectively as part of a nonrandomized unblinded study for which data on the tumor alone was previously analyzed and published.^2^ All patients underwent imaging pretreatment with both dynamic [^11^C](*R*)PK11195 TSPO PET and a comprehensive MRI protocol. Only patients with sporadic VS larger than 7.5 mm diameter in the cerebellopontine angle (>3 × full-width half-maximum, FWHM, of the PET camera) were recruited into the study and patients taking medications such as benzodiazepines or steroids that may interfere with [^11^C](*R*)PK11195 binding were excluded.^2^ To establish tumor growth, all recruited patients had undergone at least 2 MRI acquisitions, and in 2 patients this included the study MRI itself. Following a review of all available MR imaging by a multidisciplinary team of neuroradiologists and skull base surgeons, tumors were classified as static or growing. This growth classification reflected clinical decision-making, with growing tumors being recommended for microsurgery or SRS and static tumors being offered a period of radiological observation.^2^ To confirm different tumor cohort behavior, volumetric measurements of tumor size were made for both the study MRI scan and the preceding clinical scan using semi-automatic segmentation and the BrainLab iPlan software (BrainLAB). The study complied with ethical standards and received approval from the Greater Manchester North-West Research Ethics Committee (REC reference 15/NW/0429).

### [^11^C](R)PK11195 PET and MR Imaging

[^11^C](*R*)PK11195 PET scans were acquired using a high-resolution research tomograph (CTI/Siemens Molecular Imaging). Data were acquired for 60 min after [^11^C](*R*)PK11195 injection. Dynamic PET images were reconstructed with a 3D iterative algorithm; ordered subset expectation maximization (OSEM-3D) using 12 iterations and 16 subsets.^25^ The reconstructed PET images had a voxel size of 1.22 mm × 1.22 mm × 1.22 mm but to reduce image noise on the voxel level post reconstruction 3D Gaussian smoothing filters (2 mm and 4 mm FWHM kernels) were applied to the resulting images. In contrast to second-generation TSPO tracers, where binding is complicated by a single nucleotide polymorphism (rs6971) in the TSPO gene, genetic polymorphism-related differences in binding affinity have not been seen with first-generation TSPO ligands such as ^11^C-(*R*)-PK11195.^26–28^ Evaluation of single nucleotide polymorphism (rs6971) in the TSPO gene was therefore not required as part of this initial prospective study.

To ensure there was no coexisting structural abnormality within the brain and for delineation of regions of interest (ROI) for PET analysis, all patients underwent whole-brain coverage 3D T1-weighted imaging before and after administration of a gadolinium-based contrast agent (gadoterate meglumine; Dotarem, Guerbet S.A.). All MRI was performed using a 1.5-tesla Philips Achieva (Philips) (see Supplementary Methods for further details)

### [^11^C](R)PK11195 PET Analysis

A summed [^11^C](*R*)PK11195 PET image was created from the 2 mm smoothed frames and used for coregistration of the T1-weighted MRI into PET space using FSL FLIRT.^29^ The coregistered T1W image was segmented into GM and WM probability maps using SPM12,^30^ and the GM and WM probability maps were then thresholded (>0.5) to exclude erroneous non-GM or non-WM voxels respectively.^22,31^ For region of interest (ROI) analysis the brain Hammers atlas^32^ was then warped into each individual PET space. For atlas-based analysis, global brain cortex (defined as gray matter only, GM), global brain WM, and whole-brain ROI (defined as gray and white matter, GM and WM) were created by combining brain regions from the thresholded segmentation images. Ipsilesional and contralesional ROIs were also created from the GM only, WM only (excluding brainstem and bilateral commissural fibers such as the corpus callosum) and whole-brain segmented regions.

Parametric images of [^11^C](*R*)PK11195 binding potential (BP_ND_, the ratio of specifically bound radiotracer over the nondisplaceable radiotracer in tissue at equilibrium), were generated using the simplified reference tissue model with cerebellar GM time-activity curve as a pseudo reference tissue input function.^2,22,33^ Maps of distribution volume ratio (DVR = BP_ND_ + 1) were also created and DVR values were obtained by projecting the atlas-based ROIs onto the [^11^C](*R*)PK11195 parametric maps.

For deriving tumoral DVR estimates, individual tumors were manually delineated on the coregistered pretreatment T1W postcontrast image using Analyze version 11 (Biomedical Imaging Resource, Mayo Clinic) to create a tumor object mask. Care was taken to avoid partial volume effects from surrounding cerebrospinal fluid (CSF) and the object mask was projected onto the parametric BP_ND_ and DVR map so that mean tumoral DVR values could be derived.

### Voxel-Wise Analysis

A voxel-wise analysis of parametric [^11^C](*R*)PK11195 DVR images was additionally performed within SPM12 to assess the difference within normal-appearing brain regions between growing and static VS patients. The tumor was first removed from the T1-weighted MRI and [^11^C](*R*)PK11195 DVR images. In patients with a left-sided tumor, the individual structural T1-weighted MRI and [^11^C](*R*)PK11195 DVR were first left-right flipped so that in all patients, irrespective of tumor side, the hemisphere ipsilateral to the tumor was on the right side of the image. Preprocessing steps as part of the voxel-wise analysis included: segmentation, normalization to a symmetric T1 image template (modified from the standard MNI template within SPM12), and smoothing, and further details are provided in Supplementary Methods.

The voxel-wise comparison between growing and static VS groups was performed using a 2-sample *t*-test, with tumor laterality and patient age as covariates. Laterality was included in the SPM model as a covariate to account for potential [^11^C](*R*)PK11195 DVR asymmetry in the brain. An explicit mask was used to restrict the analyses to voxels within the brain only. Due to the sample size and to increase the sensitivity of the analysis, the threshold of *P* < .001 under uncorrected statistics at the voxel level was applied. However, only clusters surviving multiple comparison corrections at a family-wise error significance level of *P* < .05 are reported.

### Tissue Analysis

To evaluate the relationship between intratumoral inflammation/Iba1^+^ macrophage abundance and TSPO expression within normal-appearing brain regions and the tumor itself, previously analyzed matched tissue data from 8 VSs that had undergone surgical resection were included. This cohort included tumor tissue from 5 patients with growing VS; and 3 patients with static VS who opted for surgery despite an initial recommendation of radiological observation. Detailed protocols and a description of the semi-automatic thresholding method used for Iba1^+^ cell percentage quantification is described in prior publications.^2,14^ Ethical approval was in place for tissue analyses (REC reference 15/NW/0429).

### Statistical Analysis

GraphPad Prism 9.02 (Graphpad Inc) was used for all atlas-based statistical analyses. For nonparametric data (eg tumor volume, annual % tumor growth rate) the Mann–Whitney *U*-test was used and categorical variables (eg biological sex, the proportion of patients with nonaudiological symptoms or associated medical comorbidities) were compared using Fisher’s exact test. The normality of the regional [^11^C](*R*)PK11195 DVR values was inspected with Shapiro–Wilk test and 2 samples *t*-test was used to compare differences in [^11^C](*R*)PK11195 DVR between the 2 VS growth groups. The level of significance was set at *P* < .05. The correlation of [^11^C](*R*)PK11195 DVR in the normal brain with tumoral [^11^C](*R*)PK11195 DVR, VS volume, annual % tumor growth rate, and tumoral tissue Iba1^+^ macrophage density (mean Iba1%) were reported using Pearson’s correlation coefficient (*r*).

## Results

### Patient Population

Demographic information and tumor growth classification for the 15 patients is presented in Table 1. The mean age was 57 years (range 26–76 years) and 9 patients were female, and there was no significant difference in either biological sex (Fisher’s exact test, *P* = .32) or mean age between the 2 VS groups (unpaired *t*-test, *P* > .05). Seven tumors were classified as growing and 8 as static by the MDT. Confirmatory volumetric measurements demonstrated that growing tumors were significantly larger median tumor volume 3.50 vs. 1.02 cm^3^, *P* = .002 and displayed a significantly higher median annual % growth rate compared with static tumors (41.4 vs. 2.59 %, *P* = .008, Mann–Whitney *U*-test). All tumors were unilateral and 10 were right-sided. All patients had a history of unilateral sensorineural hearing loss with or without associated tinnitus and 3 patients had a history of imbalance and/or vertigo. There was 1 patient with a history of depression, but there were no other documented neurological or neuropsychiatric symptoms or signs. No patients were taking steroids or benzodiazepines, and there was no significant difference between the 2 VS groups in the proportion of patients with either nonaudiological symptoms (*P* > .05) or associated medical comorbidities thought to affect brain TSPO expressions such as hypertension, type II diabetes and depression (*P* > .05, Fisher’s exact test).

**Table 1.** Demographics and Tumor Growth Status for 15 Study Participants

Patient	Tumor Growth Status	Age (years)	Tumor Laterality	Presenting Symptoms	Medical Comorbidities	Pretreatment Tumor Volume (cm^3^)	Treatment
1	Growing	32.3	Left	Tinnitus, SNHL	Nil	2.82	Surgery
2	Growing	25.7	Right	Tinnitus, SNHL	Nil	5.83	Surgery
3	Growing	74.9	Right	Tinnitus, SNHL	T2DM	1.54	SRS
4	Growing	64.6	Right	SNHL	Previous contralateral mastoiditis	1.43	SRS
5	Growing	54.3	Left	Tinnitus, SNHL	HTN	4.24	Surgery
6	Growing	54.8	Left	Tinnitus, SNHL	T2DM	6.76	Surgery
7	Growing	39.0	Right	Tinnitus, SNHL, imbalance, headaches	Nil	3.50	Surgery
8	Static	58.8	Right	SNHL	T2DM, HTN	1.32	Radiological FU
9	Static	60.8	Right	Tinnitus, SNHL	Nil	1.00	Radiological FU
10	Static	56.3	Right	Tinnitus, SNHL, imbalance, vertigo	Depression	0.49	Surgery
11	Static	76.1	Left	Tinnitus, SNHL	Nil	1.53	Radiological FU
12	Static	61.1	Right	Tinnitus, SNHL	Hashimoto’s, OA, hypothyroidism, Raynaud’s, prediabetes	0.82	Surgery
13	Static	73.9	Right	SNHL, imbalance	Hypothyroidism	1.22	Radiological FU
14	Static	63.0	Left	SNHL	Asthma	0.70	Surgery
15	Static	58.2	Right	Tinnitus, SNHL	HTN	1.04	Radiological FU

FU = follow up; HTN = hypertension; Nil = no other known medical comorbidity; OA = osteoarthritis; SNHL = sensorineural hearing loss; SRS = stereotactic radiosurgery; T2DM = type 2 diabetes.

### Global Differences in Brain TSPO Expression

To initially confirm suitability of the cerebellar GM as a pseudo reference region, cerebellar GM [^11^C](R)PK11195 standardized uptake values (SUV_40–60min_), a semi-quantitative model-free approach, were compared between growing and static VS. This demonstrated no significant difference in cerebellar GM [^11^C](R)PK11195 SUV_40–60min_ between growing and static VS groups (*P* > .05, unpaired *t*-test Supplementary Figure 1).

**Figure 1.**
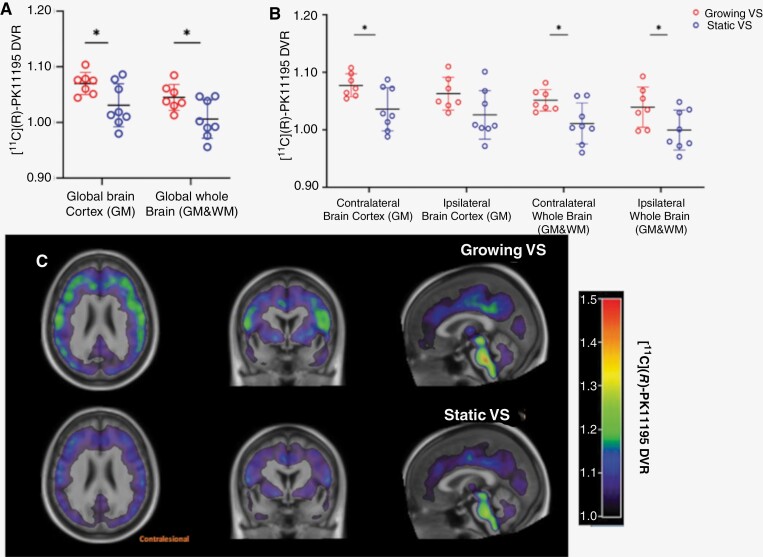
[^11^C](*R*)PK11195 DVR in normal-appearing brain. (A) Dot plot demonstrating [^11^C](*R*)PK11195 DVR values in global brain cortex (GM) and whole brain (GM & WM). Red circles (first column) represent growing VS; blue (second column) represents static VS. Horizontal black lines represent the mean ± 1 SD. Asterisk (*) indicates *P* < .05; unpaired 2-sample *t*-test. (B) Dot plot demonstrating breakdown of [^11^C](*R*)PK11195 DVR values in ipsilesional and contralesional brain cortex (GM) and whole brain (GM & WM). (C) Summed [^11^C](*R*)-PK11195 DVR image combining DVR values from the growing (*top row*) and static VS (*bottom row*). Summed [^11^C](*R*)PK11195 DVR image shown overlaid on average T1W image. In all patients, the hemisphere ipsilateral to the tumor (ipsilesional) is shown on the right side with contralateral brain (contralesional) shown on the left. Note in patients with growing VS the increased [^11^C](*R*)PK11195 DVR within supratentorial GM and WM, and the increased [^11^C](*R*)PK11195 DVR within contralesional vs. ipsilesional brain areas.

[^11^C](*R*)PK11195 DVR values in global brain cortex (GM) and whole brain (GM & WM) are presented in Figure 1A. Patients with growing VS demonstrated significantly higher [^11^C](*R*)PK11195 DVR in global brain cortex (GM, 1.070 vs. 1.031, *P* = .03) and whole brain (GM & WM, 1.045 vs. 1.006, *P* = .03) compared with static tumors, reflecting increased brain TSPO expression.

[^11^C](*R*)PK11195 DVR values in ipsilesional and contralesional brain regions are presented in Figure 1B. Like the global DVR, growing VS patients displayed significantly higher [^11^C](*R*)PK11195 DVR values within contralesional GM cortex compared to patients with static tumors (1.077 vs. 1.036, *P* = .02). Both contralesional (1.077 vs. 1.011, *P* = .02) and ipsilesional (1.040 vs. 0.999, *P* = .04) whole brain (GM & WM) in the growing VS patients also demonstrated significantly higher [^11^C](*R*)PK11195 DVR compared to patients with static VS. In Supplementary Figure 2 [^11^C](R)PK11195 DVR values in global, ipsilesional and contralesional atlas-defined WM regions are also presented. While growing VS demonstrated higher [^11^C](R)PK11195 DVR in global (WM, 1.033 vs. 1.002, *P* = .12,), contralesional (1.026 vs. 0.9958, *P* = .19), and ipsilesional WM (1.039 vs. 1.008, *P* = .11, unpaired *t-*test) compared to patients with static tumors these differences did not reach statistical significance.

A summed [^11^C](*R*)PK11195 DVR image combining DVR values from the entire growing and static VS cohort, respectively is shown in Figure 1C. Qualitatively, the images demonstrate marked differences in TSPO expression between patients with growing and static VS within structurally normal supratentorial brain regions.

### Brain TSPO Expression, Tumor Growth, and Intratumoral Inflammation

**Figure 2.**
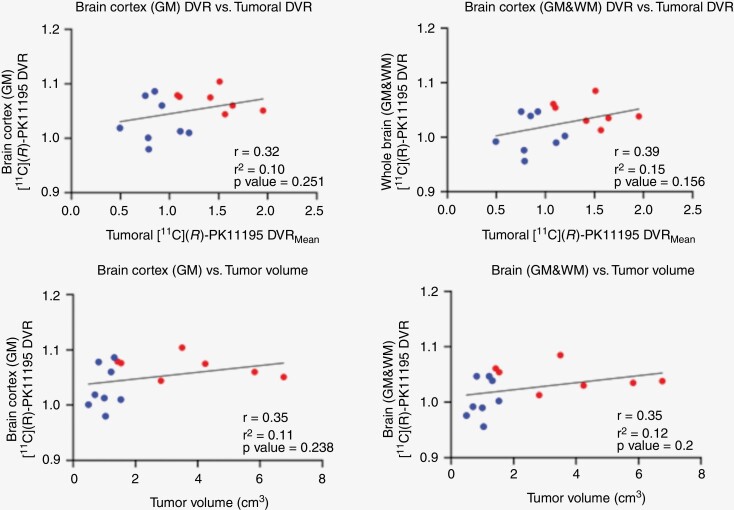
Correlation of brain TSPO expression with tumor size and tumor [^11^C](*R*)PK11195 DVR. Correlation results of [^11^C](*R*)-PK11195 DVR in global brain cortex (GM) and global whole brain (GM & WM) against mean tumoral [^11^C](*R*)PK11195 DVR and tumor volume (cm^3^). Red circles represent growing VS; blue represent static VS. Pearson’s product–moment correlation coefficient (*r*) and adjusted *r*^2^ estimates reported.

Correlations results are shown in Figure 2 and Table 2. A positive but statistically insignificant correlation was observed between global whole-brain (GM & WM, *r* = 0.39, *P* = .16) and cortical [^11^C](R)PK11195 DVR (GM, *r* = 0.32, *P* = .25) with mean tumoral [^11^C](R)PK11195 DVR. A positive but statistically nonsignificant (*P* > .05) correlation was also observed between global whole-brain (GM & WM) and cortical [^11^C](*R*)PK11195 DVR (GM) with tumor volume and % annual tumor growth rate, with larger faster-growing tumors trending to greater brain TSPO expression. A positive correlation was also observed between individual contralesional and ipsilesional brain areas with tumor [^11^C](R)-PK11195 DVR and tumor volume, but these correlations did not reach statistical significance (*P* > .05, Table 2 and Supplementary Figures 3 and 4).

**Table 2.** Correlation Analysis of [^11^C](*R*)PK11195 DVR in Whole Brain Cortex (GM) and Whole Brain (GM & WM) Against Mean and Max Tumor [^11^C](*R*)-PK11195 DVR, Tumor Volume (cm^3^), And % Tumor Growth Rate

Correlation tests (Pearson)	*r*	*r* ^2^	CI 95%	*P* value
**Brain (GM & WM) vs. Tumoral DVR**_**Mean**_ Ipsilesional brain (GM & WM) vs. tumoral DVR_Mean_ Contralesional brain (GM & WM) vs. tumoral DVR_Mean_	0.39	0.15	–0.16–0.75	.156
	0.35	0.13	–0.19–0.73	.196
	0.43	0.18	–0.11–0.77	.110
**Brain (GM & WM) vs. Tumoral DVR**_**Max**_ Ipsilesional brain (GM & WM) vs. tumoral DVR_Max_ Contralesional brain (GM & WM) vs. tumoral DVR_Max_	0.44	0.20	–0.09–0.78	.097
	0.43	0.18	–0.11–0.77	.111
	0.46	0.21	–0.07–0.79	.084
**Brain (GM & WM) vs. tumor volume** Ipsilesional brain (GM & WM) vs. tumor volume Contralesional brain (GM & WM) vs. tumor volume	0.35	0.12	–0.20–0.73	.200
	0.29	0.09	–0.26–0.70	.287
	0.40	0.16	–0.14–0.76	.135
**Brain (GM & WM) vs. % Tumor growth rate** Ipsilesional brain (GM & WM) vs. % tumor growth rate Contralesional brain (GM & WM) vs. % tumor growth rate	0.26	0.07	–0.29–0.68	.354
	0.22	0.05	–0.33–0.66	.433
	0.30	0.09	–0.26–0.70	.286
**Brain cortex (GM) vs. Tumoral DVR**_**Mean**_ Ipsilesional brain cortex (GM) vs. tumoral DVR_Mean_ Contralesional brain cortex (GM) vs. tumoral DVR_Mean_	0.32	0.10	–0.23–0.71	.251
	0.25	0.06	–0.30–0.67	.375
	0.36	0.13	–0.18–0.74	.182
**Brain cortex (GM) vs. Tumoral DVR**_**Max**_ Ipsilesional brain cortex (GM) vs. tumoral DVR_Max_ Contralesional brain cortex (GM) vs. tumoral DVR_Max_	0.39	0.16	–0.15–0.75	.145
	0.34	0.11	–0.21–0.72	.219
	0.42	0.18	–0.12–0.77	.117
**Brain cortex (GM) vs. tumor volume** Ipsilesional brain cortex (GM) vs. tumor volume Contralesional brain cortex (GM) vs. tumor volume	0.35	0.11	–0.23–0.72	.238
	0.24	0.06	–0.32–0.67	.399
	0.39	0.15	–0.15–0.75	.148
**Brain cortex (GM) vs. % tumor growth rate** Ipsilesional brain cortex (GM) vs. % tumor growth rate Contralesional brain cortex (GM) vs. % tumor growth rate	0.28	0.10	–0.27–0.69	.311
	0.20	0.04	–0.34–0.65	.464
	0.33	0.11	–0.22–0.72	.225

Pearson’s product–moment correlation coefficient (*r*) was reported along with the results of linear regression (*r*^2^ estimates).

DVR = distribution volume ratio, GM = gray matter, WM = white matter, CI = confidence intervals.

**Figure 3.**
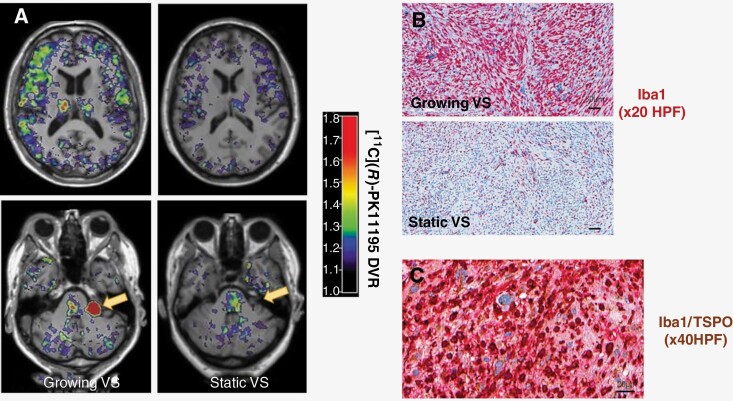
Brain TSPO expression and intratumoral inflammation. (A) Representative [^11^C](*R*)PK11195 DVR images (overlaid on T1W images) from a patient with a growing and static VS, respectively. *Bottom row:* Axial image through cerebellopontine angle shows increased [^11^C](*R*)PK11195 DVR within growing VS relative to static tumor (arrow). *Top row:* Axial image through supratentorial brain regions shows increased [^11^C](*R*)PK11195 DVR within contralesional GM and WM in the patient with a growing VS. (B) Representative tumor tissue immunostains (Iba1 = red) from the growing and static VS shown in panel B. Note the high abundance of Iba1^+^ macrophages within the growing VS relative to the static tumor (immunoperoxidase, ×20 HPF). C: Double immunostained (Iba1 = red /TSPO = brown) image sections from the growing VS shown in panel A and B demonstrating colocalization of TSPO expression within the cytoplasm of Iba1^+^ macrophages and demonstrating macrophages as the principal source of TSPO binding (immunoperoxidase, ×40 HPF).

In keeping with earlier published tumoral [^11^C](R)-PK11195 BP_ND_ values,^2^ a significant positive correlation of intratumoral inflammation/macrophage abundance (Iba1^+^ cell %) with mean tumoral [^11^C](*R*)PK11195 DVR (*r* = 0.87, *P* = .005) was seen. A positive correlation was also observed between intratumoral Iba1^+^ cell % with global brain cortex (GM) and whole brain (GM & WM) [^11^C](*R*)PK11195 DVR, but these did not reach statistical significance (GM, *r* = 0.40, *P* = .33; GM & WM, *r* = 0.37, *P* = .36, respectively). Representative imaging and immunohistochemistry stained tissue sections from a patient with a fast growing VS and static VS, respectively are also shown in Figure 3 and demonstrate the association between tumor growth, intratumoral inflammation, and elevated global TSPO expression within both the ipsilesional and contralesional supratentorial brain.

### Voxel-Based Cluster Analysis

**Figure 4.**
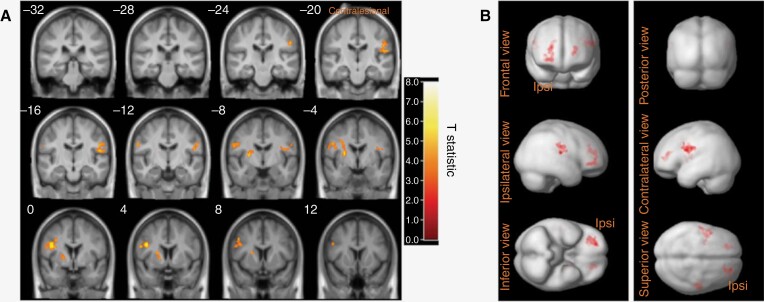
Voxel-wise SPM analysis of [^11^C](*R*)PK11195 DVR. The location of significant clusters (FWE *P* < .05) with voxel-wise differences between static and growing VS are shown. The color bar values indicate the value of the *T*-statistic. Clusters shown overlaid on coronal template T1W images in MNI space (panel A). 3D brain renders also shown with significant clusters shown in red (panel B). In patients with a left-sided tumor, the individual structural T1-weighted MRI and parametric [^11^C](*R*)PK11195 DVR images were first left-right flipped. In all patients, the hemisphere ipsilateral to the tumor (ipsilesional) is therefore shown on the right side. Clusters of increased [^11^C](*R*)PK11195 binding were observed in the ipsilesional postcentral gyrus, ipsilesional prefrontal cortex, contralesional precentral gyrus, and contralesional prefrontal cortex in patients with growing VS compared to patients with static tumors. Parametric [^11^C](*R*)PK11195 DVR images were smoothed using a 3-dimensional 6-mm FWHM Gaussian filter before statistical analysis and the threshold of *P* < .05 under FWE corrected statistics at the cluster level (FWEc) was applied.

Differences between growing and static VS groups are shown in Figure 4. Significant clusters of increased [^11^C](*R*)PK11195 binding (FWE *P* < .05) were observed in the ipsilesional postcentral gyrus, ipsilesional prefrontal cortex, contralesional precentral gyrus, and contralesional prefrontal cortex in patients with growing VS compared to patients with static tumors. The relevant *T-*statistic for each significant voxel cluster is shown in Supplementary Table 1.

## Discussion

In this study we have investigated, for the first time, TSPO expression in the normal-appearing brain of patients with VS. Our results demonstrate that in patients with growing VS, there is widespread TSPO upregulation within normal-appearing brain regions, indicative of microglial activation. An included dedicated voxel cluster analysis demonstrated that this TSPO upregulation predominantly occurs within nonauditory frontal brain regions and may therefore impact on both nonaudiovestibular symptoms and cognitive functioning within affected patients.

There is growing evidence that despite the histologically benign, noninvasive nature of VS, these tumors can have widespread effects remote to the tumor both within the brain and blood. Recent studies have for example demonstrated elevated neutrophil-to-lymphocyte ratio, a systemic marker of inflammation, in patients with growing VS,^34^ and decreased progression-free survival in patients with a high baseline serum CRP (≥3.14 mg/dL) level at the time of VS diagnosis.^35^ In keeping with the dense infiltration of tumor-associated macrophages seen in growing VS, studies have also shown upregulation of numerous monocytic cytokines in the plasma of patients with growing VS.^14^ Evidence that sporadic VS can have widespread effects remote to the tumor also comes from studies of SNHL in affected patients, with emerging evidence that direct damage to cochlea hair cells may occur through factors secreted into the CSF or blood by the tumor such as MMP-14 and the pro-inflammatory cytokine TNFα.^4,5,36^ There has to date, however, been no assessment of VS induced inflammatory changes within the brain parenchyma itself.

Previous diffusion tensor imaging (DTI) studies in patients with unilateral VS have demonstrated evidence of WM damage in ascending auditory pathways remote to the tumor.^8,13,37^ In 1 previous DTI study, increased apparent diffusion coefficient (ADC) and decreased fractional anisotropy (FA) values were observed in contralateral ascending auditory pathway structures in VS patients when compared to controls,^13^ but without significant changes within the primary auditory cortex (Heschl’s gyrus) itself. The authors hypothesized that higher ADC values might be related to microstructural changes such as loss of myelin or demyelination and that the predominance of contralateral ADC changes reflects the predominance of fibers (70–80%) that ascend contralaterally to each ear.^8,13^ Other authors have corroborated these earlier DTI findings^37^ but also demonstrated widespread changes in diffusion scalar measures (ADC and FA) beyond classical auditory areas and an association for example between cognitive changes in patients with sporadic VS and decreases in FA within frontal WM tracts (eg forceps minor).^8^

An interesting aspect of our results is that the highest [^11^C](*R*)PK11195 DVR values within growing tumors were seen in contralateral brain regions. Demyelination and remyelination are known to induce both microglial and astrocytic TSPO expression, with microglial expression predominating during the demyelination phase.^17,38,39^ It is therefore conceivable that the observed increased contralateral GM and WM TSPO expression partly reflects reactive microglial activation following disruption to ascending auditory pathways in growing tumors. The inclusion of a voxel-based cluster analysis in our study, however, also demonstrated that increased TSPO binding predominated in nonauditory brain regions including the contralesional precentral gyrus, ipsilesional postcentral gyrus, and ipsilesional and contralesional prefrontal cortex. Previous authors using voxel-based morphometry analyses of structural MRI have compared VS patients with healthy controls and reported significantly increased GM volume in the supplementary motor area, bilateral precentral gyrus, and bilateral postcentral gyrus.^12^ The authors hypothesized that increased listening effort due to VS-associated SNHL was associated with compensatory increased activity and neuroplastic reorganization in the premotor cortex.^12^ Other authors have similarly reported widespread alterations in both WM structures and GM volume in higher auditory regions,^7^ and have demonstrated an association between cognitive changes and widespread functional activity changes on fMRI, with some areas showing increased compensatory activity and other areas such as the frontal lobe showing decreased regional homogeneity indicative of functional connectivity loss.^7^ Together the results of the above studies indicate that in sporadic VS patients, cortical reorganization, and neuroplasticity are prominent, occur within both higher auditory and nonauditory processing areas, and are likely induced by both compromised sensory input due to SNHL and tinnitus and a compensatory response to declining cognitive function.^7,8,12^

Within the VS tumor microenvironment, the principal source of [^11^C](*R*)PK11195 binding has been shown through immunohistochemical studies to be TSPO-expressing blood-derived macrophages.^2^ Within the brain parenchyma, however, microglia and/or reactive astrocytes are most likely the origin of this TSPO expression.^17,19,20^ It can be hypothesized that this TSPO expression reflects a state of neuroinflammation and widespread microglial activation in response to tumor growth and/or changes in circulating pro-inflammatory chemokine expression.^14^ This activation and its demonstrated localization within prefrontal cortical areas may in part explain the cognitive deficits experience by such patients, but also may reflect the neuroplasticity and cortical reorganization identified in previous works.^7,8,12^ Microglia are normally found throughout both gray and WM in the CNS,^40–42^ but methodological reasons such as partial volume effects, high nonspecific binding within white compared to GM,^43,44^ and use of cerebellar GM as a “pseudoreference” region may all have impacted on atlas-defined WM region [^11^C](*R*)PK11195 DVR values and the observed nonstatistically significant WM differences between patients with growing and static VS.

In vivo, higher TSPO PET binding may reflect both higher TSPO expression within each individual cell and/ or overall higher cellular abundance. Previous murine and in vivo human studies in intrinsic CNS tumors such as high-grade glioma have combined advanced methods such as immunomagnetic cell sorting (MACS) after in vivo radiotracer injection^45,46^ (scRadiotracing) and 3D histology to interrogate TSPO-expressing cell populations within the tumor microenvironment, demonstrating, for example, higher TSPO binding within neoplastic tumor cells compared to tumor-associated microglia/macrophages. In studies of normal-appearing brain changes in patients with VS, however, tissue biopsy of such regions is not feasible leading to an absence of matching brain tissue through which to correlate the imaging findings. A presumed microglial source of TSPO upregulation in the brains of patients with growing VS can therefore not be fully confirmed and future in vivo studies which combine techniques such as scRadiotracing^45,46^ with animal models that accurately recapitulate the VS microenvironment,^47,48^ are required to confirm the exact cellular source of TSPO upregulation in these regions.

### Limitations

A limitation of our study is that we did not undertake a comprehensive neuropsychiatric evaluation on all recruited patients or evaluate for concomitant elevations in blood-based markers of systemic inflammation. The primary outcome measure for the initial prospective study was differences in tumoral [^11^C](*R*)PK11195 between VS growth groups and as such this preliminary study was therefore neither designed nor powered to detect subtle differences between groups regarding either their neuropsychiatric symptoms or systemic inflammation markers. Dedicated longitudinal studies which incorporate neuropsychological testing, imaging studies of TSPO expression, and systemic inflammation biomarker profiles, both before and after treatment for a VS, should therefore be undertaken to better understand the relationship between VS growth and treatment, cortical TSPO upregulation and cognitive symptoms in this patient group.

In contrast to second-generation TSPO tracers, genetic polymorphism-related differences in ^11^C-(*R*)-PK11195 binding affinity are not seen.^26–28^ Second-generation TSPO PET ligands such as ^18^F-GE180 and ^18^F-DPA-714 do however offer higher TSPO affinity, better signal-to-noise, and lower nonspecific binding. Additionally, a key limitation in the widespread clinical application of ^11^C-(*R*)-PK11195 and its applicability with advanced ex vivo methods for discriminating cellular TSPO PET binding such as scRadiotracing,^45,46^ is the tracer’s short half-life. Further human and in vivo animal studies incorporating genetic polymorphism screening and these more clinically applicable second-generation TSPO tracers should be considered to further investigate cellular TSPO expression in both tumoral and normal-appearing brain regions.

Our results provide important preliminary evidence that in patients with growing VS, there is widespread TSPO upregulation within normal-appearing brain regions, indicative of microglial activation. Within this study, we have used a cohort of patients with nongrowing (static) VS as a disease control cohort but there is, however, an unclear magnitude of [^11^C](R)PK11195 binding in VS when compared to normal brains from healthy, age-matched populations. Selection of true healthy individuals as a control group can be challenging as even in the absence of known disease, undiagnosed chronic systemic inflammatory states that can influence brain TSPO expression such as atherosclerosis, dyslipidemia, and diabetes can exist.^21^ Larger prospective studies with full age and gender-matched controls, which have been rigorously screened for other recognized causes of brain TSPO upregulation, are therefore required to robustly evaluate differences in [^11^C](R)PK11195 binding between VS and healthy control populations.

## Conclusions

The presented results demonstrate for the first time that in patients with growing sporadic VS there is increased [^11^C](*R*)PK11195 binding indicative of microglial activation and inflammation within apparently normal-appearing supratentorial brain regions. This upregulation predominantly occurs within nonauditory frontal brain regions potentially impacting nonaudiovestibular symptoms within affected patients. These results highlight the need for further studies interrogating the cellular origin of the demonstrated increased TSPO binding within the demonstrated brain regions and the role of widespread neuroinflammation in driving VS symptomatology.

## Supplementary Material

vdae094_suppl_Supplementary_Tables_S1

vdae094_suppl_Supplementary_Figures_S1-S4

vdae094_suppl_Supplementary_Materials
