# Supplementary material for: Evidence for inflammation in normal-appearing brain regions in patients with growing sporadic vestibular schwannoma: A PET study
Source: Neurooncol Adv. 2024 Jun 8;6(1):vdae094. doi: 10.1093/noajnl/vdae094 (PMC11221070; doi:10.1093/noajnl/vdae094)
Supplement: vdae094_suppl_Supplementary_Tables_S1 [file vdae094_suppl_supplementary_tables_s1.docx]

**Supplementary Table S1: Significant voxel-wise clusters, anatomical location, and T statistics**

|  | **Cluster level** | | | **Peak level T-value** | **Structure** | |
| --- | --- | --- | --- | --- | --- | --- |
| **Analysis** | **Number of voxels** | **Cluster p(FWE-corr)** | **Cluster p(unc)** |  |  |  |
| **Growing VS > Static VS** | 1467.00 | 1.30E-06 | 6.06E-08 | 10.33 | Contralesional Cerebrum / Frontal Lobe / Inferior Frontal Gyrus / Precentral Gyrus |  |
|  |  |  |  | 6.96 |  |  |
|  |  |  |  | 6.46 |  |  |
|  | 440.00 | 1.25E-02 | 5.83E-04 | 8.49 | Contralesional Cerebrum / Frontal Lobe |  |
|  |  |  |  | 6.59 |  |  |
|  |  |  |  | 6.21 |  |  |
|  | 1273.00 | 5.84E-06 | 2.71E-07 | 8.17 | Ipsilesional Cerebrum / Frontal Lobe / Superior Frontal Gyrus |  |
|  |  |  |  | 7.30 |  |  |
|  |  |  |  | 7.05 |  |  |
|  | 717.00 | 7.16E-04 | 3.32E-05 | 7.15 | Ipsilesional Cerebrum / Parietal Lobe / Postcentral Gyrus / Frontal Lobe / Precentral Gyrus |  |
|  |  |  |  | 6.46 |  |  |
|  |  |  |  | 5.90 |  |  |

Vestibular schwannoma (VS), FWE-corr = family-wise error correction, unc = uncorrected
