# Supplementary material for: Evidence for inflammation in normal-appearing brain regions in patients with growing sporadic vestibular schwannoma: A PET study
Source: Neurooncol Adv. 2024 Jun 8;6(1):vdae094. doi: 10.1093/noajnl/vdae094 (PMC11221070; doi:10.1093/noajnl/vdae094)

**Supplementary figures**

**Supplementary Figure S1: [^11^C](R)PK11195 standardised uptake values (SUV 40-60min) cerebellar pseudoreference grey matter (GM) region**


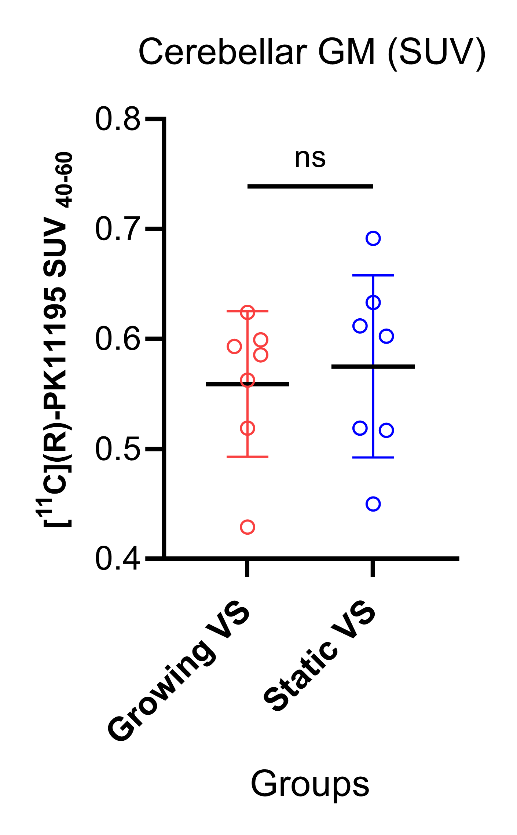
The horizontal black lines represent the mean plus/minus one-stander deviations; “ns” indicates not significant from the unpaired t-test between growing and static VS groups.

**Supplementary Figure S2:** **[^11^C](R)PK11195 distribution volume ratio (DVR) in normal appearing brain.**

A: Dot plot demonstrating the [^11^C](R)PK11195 DVR values in the whole brain (GM&WM), global brain cortex (GM) and global white matter (WM). Red circles represent growing VS, and blue represents static VS.

B: Dot plot demonstrating the breakdown of [^11^C](R)PK11195 DVR values in ipsilesional and contralesional whole brain (GM&WM), brain cortex (GM), and white matter (WM).

Horizontal black lines represent the mean plus/minus one standard deviation. The symbol “ns” indicates no significant results from the unpaired t-test, whereas the asterisk (*) indicates p < 0.05; unpaired two-sample t-test.

**
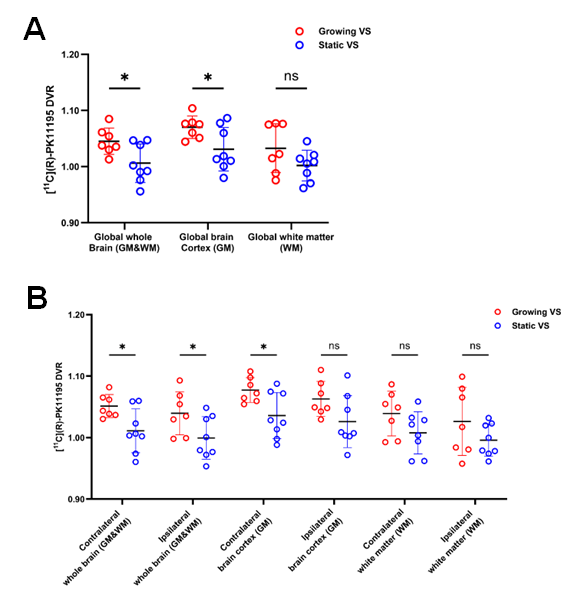
**

**Supplementary Figure S3:** Correlation results of [^11^C](R)PK11195 DVR in global whole brain (GM&WM) and brain cortex (GM) (and their breakdown into ipsilesional and contralesional) DVR against mean tumour [^11^C](R)PK11195 DVR.

Pearson’s product-moment correlation coefficient (r) adjusted r^2^ estimates and p-values are reported.


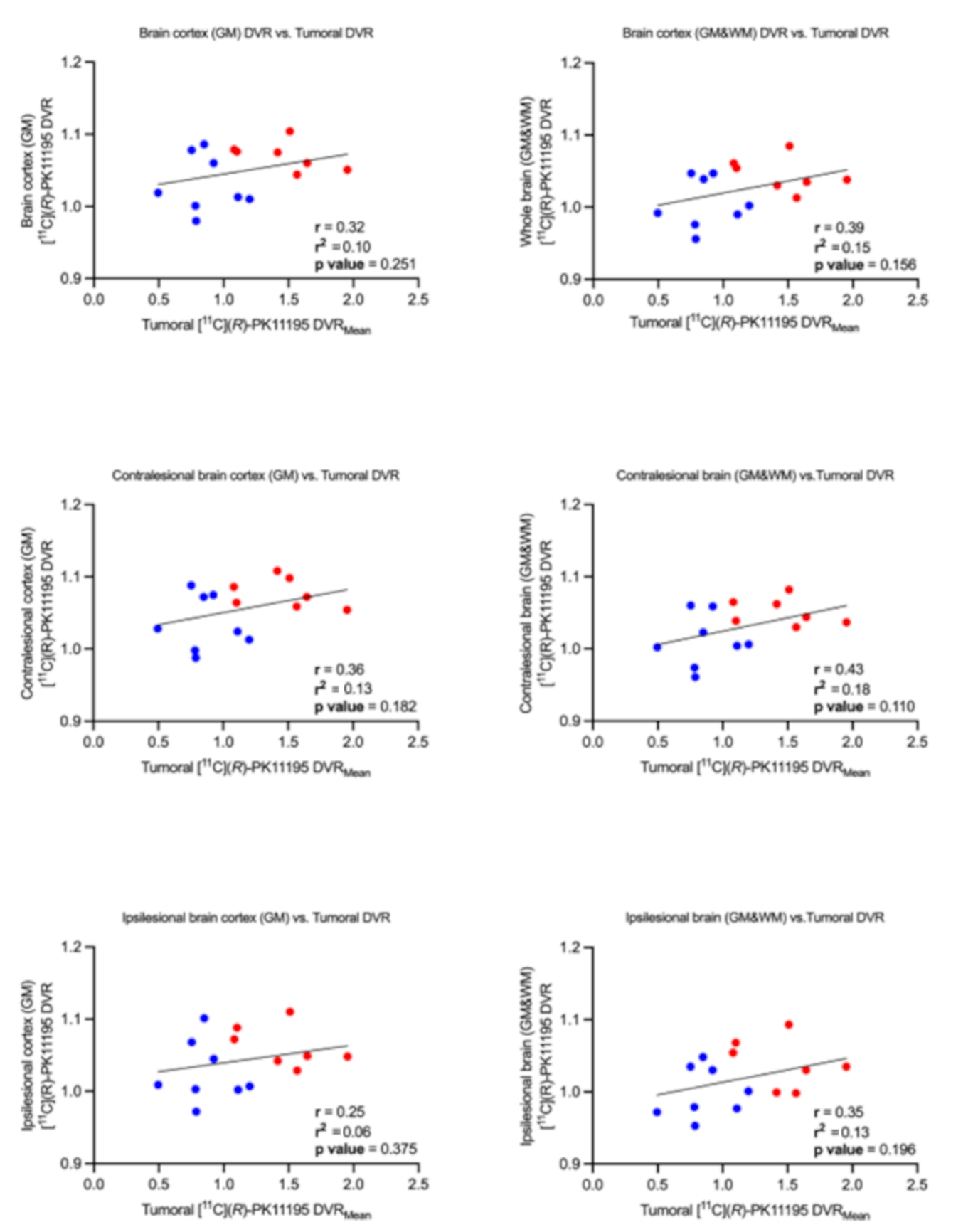


**Supplementary Figure S4**: Correlation results of [^11^C](R)PK11195 DVR in global whole brain (GM&WM) and brain cortex (GM) )and their breakdown into ipsilesional and contralesional) DVR against tumour volume (cm^3^).

Pearson’s product-moment correlation coefficient (r) adjusted r^2^ estimates and p-values are reported.


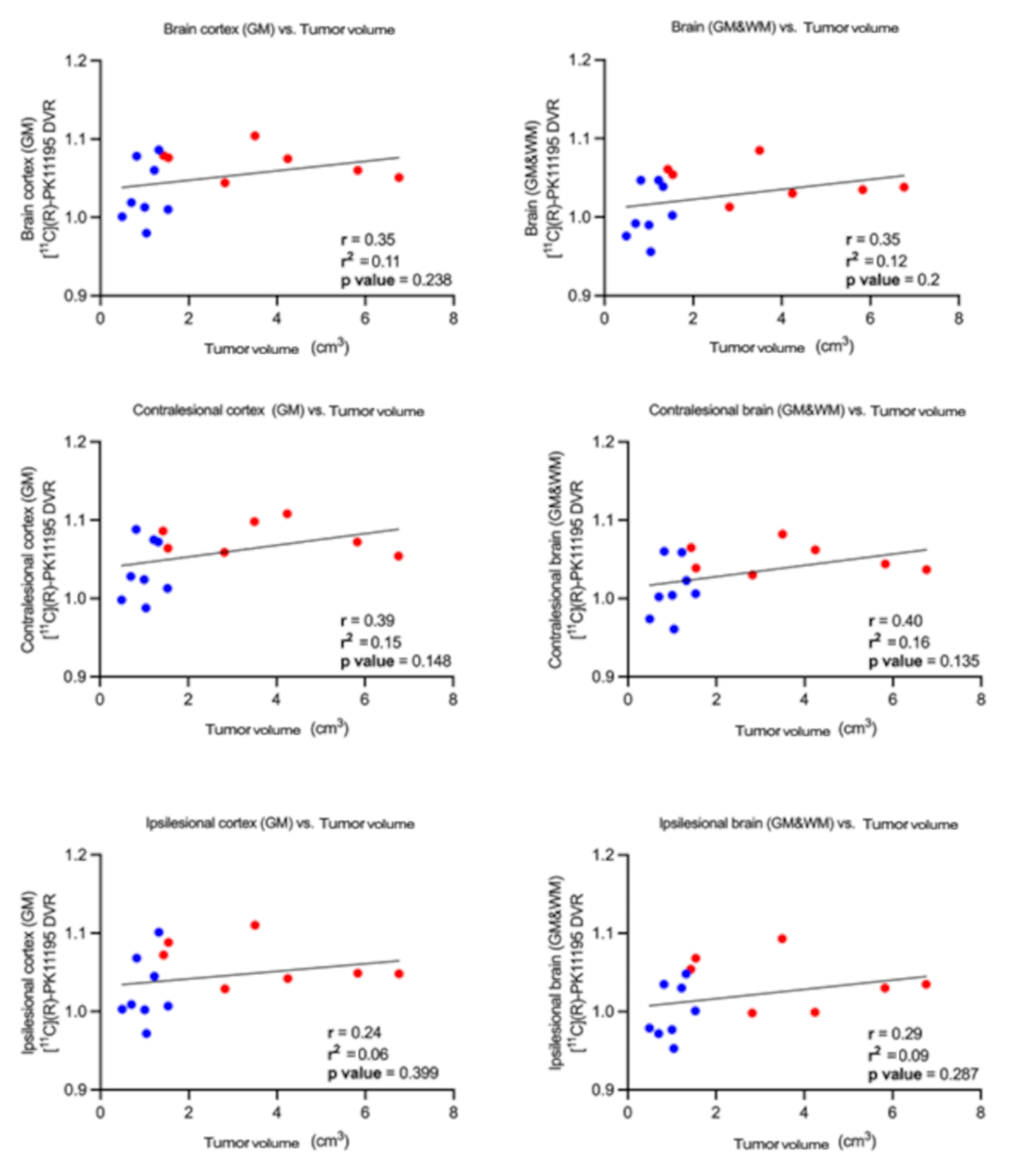

Supplement: vdae094_suppl_Supplementary_Figures_S1-S4 [file vdae094_suppl_supplementary_figures_s1-s4.docx]
