# Supplementary material for: Evidence for inflammation in normal-appearing brain regions in patients with growing sporadic vestibular schwannoma: A PET study
Source: Neurooncol Adv. 2024 Jun 8;6(1):vdae094. doi: 10.1093/noajnl/vdae094 (PMC11221070; doi:10.1093/noajnl/vdae094)
Supplement: vdae094_suppl_Supplementary_Materials [file vdae094_suppl_supplementary_materials.docx]

## Supplementary Methods

## Structural Magnetic Resonance Imaging (MRI)

All recruited patients underwent structural magnetic resonance imaging (MRI) using a 1.5-Tesla Philips Achieva whole body scanner and a dedicated head coil (Philips, Best, Netherlands). High spatial resolution whole brain coverage 3D T1-weighted (T1W) imaging was obtained using a gradient echo sequence. with the following acquisition parameters: repetition time (TR) 8.6 ms, echo time (TE) 3.2 ms, and slice thickness 1.2 mm. Structural T_1_-weighted MRI was utilized to ensure there was no co-existing structural abnormality within the supratentorial brain and for delineation of regions of interest (ROI) for PET image analysis. For tumour delineation, a postcontrast high resolution 3D T1W gradient echo sequence of the whole brain was also obtained at the end of the MRI protocol and following administration of a standard dose of gadolinium based contrast agent (gadoterate meglumine; Dotarem, Guerbet S.A.).

## Voxel-wise analysis

In patients with a left sided tumor, the individual structural T1-weighted MRI and [^11^C](*R*)PK11195 DVR images were first left-right flipped so that in all patients, irrespective of tumor side, the hemisphere ipsilateral to the tumor was on the right side of the image. Pre-processing steps as part of the voxel-wise analysis included: segmentation, normalization and smoothing. The individual original and flipped structural T1-weighted images were first segmented and spatially normalized to a symmetric T1 image template (modified from the standard MNI template within SPM12). The individual parametric [^11^C](*R*)PK11195 DVR images were then normalized to this symmetric common template using the transformation parameters created in the unified segmentation. The parametric [^11^C](*R*)PK11195 DVR images were then finally smoothed using a three-dimensional 6-mm FWHM Gaussian filter before statistical analysis.
